# An Idiopathic Case of Sclerosing Encapsulating Peritonitis: A Case Report

**DOI:** 10.7759/cureus.53667

**Published:** 2024-02-05

**Authors:** Meaad N Almouwalld

**Affiliations:** 1 General Surgery, Royal Commission Medical Center, Yanbu, SAU

**Keywords:** idiopathic peritonitis, exploratory laparotomy, small intestinal obstruction, abdominal cocoon syndrome, sclerosing encapsulating peritonitis

## Abstract

Sclerosing encapsulating peritonitis, also known as abdominal cocoon syndrome, is an uncommon disorder where a dense fibrous layer forms around the small intestine, causing blockage and vague abdominal complaints. Despite its infrequency, diagnosing and treating this condition is challenging due to its indistinct symptoms and the complex nature of its treatment. This report discusses a 55-year-old female with no notable medical history who experienced progressive abdominal pain and weight loss. Initial laboratory tests revealed mild normocytic anemia and raised levels of inflammatory markers. A computed tomography (CT) scan demonstrated "cocoon-like" encapsulation of the small intestines. After ruling out infectious, neoplastic, and autoimmune factors, the patient was diagnosed with idiopathic sclerosing encapsulating peritonitis. The treatment strategy began with conservative measures, including total parenteral nutrition and antibiotics, but eventually required surgical intervention due to ongoing symptoms. Postoperatively, the patient recovered well, showing significant symptom relief and weight gain at a six-month checkup. This case emphasizes the need to consider sclerosing encapsulating peritonitis when diagnosing unexplained abdominal symptoms, especially when no typical risk factors are present.

## Introduction

Sclerosing encapsulating peritonitis, also known as abdominal cocoon syndrome, is a rare condition characterized by the development of a thick fibrous membrane that encases the small bowel, leading to intestinal obstruction and a variety of nonspecific abdominal symptoms [1]. Although it has been associated with peritoneal dialysis, previous abdominal surgery, tuberculosis, and certain autoimmune conditions, sclerosing encapsulating peritonitis remains an entity with an elusive etiology in many cases [1-3]. The idiopathic form of the disease, where no underlying cause can be identified, presents a particular challenge for diagnosis and management [2,3].

Despite its rarity, sclerosing encapsulating peritonitis is of significant clinical interest due to the diagnostic and therapeutic challenges it poses. The nonspecific nature of its clinical presentation often leads to a delay in diagnosis, with many patients undergoing extensive workups before reaching the correct diagnosis. Imaging studies, particularly computed tomography (CT), play a crucial role in identifying the characteristic features of sclerosing encapsulating peritonitis [1]. Treatment strategies vary from conservative management with nutritional support and anti-inflammatory medications to surgical intervention when bowel obstruction occurs [3,4]. The complexity of sclerosing encapsulating peritonitis calls for a multidisciplinary approach to optimize outcomes for affected individuals.

## Case presentation

A 55-year-old female with a six-month history of progressive abdominal distension, intermittent abdominal pain, and a noticeable weight loss of 10 kg presented to the outpatient department. Abdominal pain was described as a dull ache that was diffusely located and occasionally exacerbated by eating. There was no history of nausea, vomiting, change in bowel habits, or fever. Her past medical history was unremarkable, with no history of previous surgeries, no history of tuberculosis, and no chronic medication use. She had no known allergies. Her family history was noncontributory.

Physical examination revealed a moderately distended abdomen with diffuse tenderness but no palpable masses or hepatosplenomegaly. There was a notable absence of shifting dullness. Bowel sounds were present and normal. Other systemic examinations were within normal limits.

Given the clinical presentation, an extensive workup was initiated to elucidate the cause of her symptoms. Initial laboratory investigations included a complete blood analysis, which revealed mild normocytic anemia with a hemoglobin level of 11.2 g/dL. White blood cell and platelet counts were within normal ranges. Biochemical profiles, including liver function test results, renal function test results, and electrolyte levels, were all within normal limits. Inflammatory markers were elevated, with a C-reactive protein level of 45 mg/L (normal: <5 mg/L) and an erythrocyte sedimentation rate of 55 mm/hour (normal: 0-20 mm/hour). Tumor marker levels were within the normal reference ranges. Serological tests for autoimmune diseases and viral hepatitis were negative (Table 1).

**Table 1:** Comprehensive laboratory investigations and their results

Laboratory Test	Result	Normal Range
Complete Blood Count		
Hemoglobin	11.2 g/dL	12.0-15.5 g/dL
White Blood Cell Count	7,200/μL	4,500-11,000/μL
Platelet Count	250,000/μL	150,000-450,000/μL
Liver Function Tests		
Alanine Aminotransferase	30 units/L	7-56 units/L
Aspartate Aminotransferase	25 units/L	10-40 units/L
Alkaline Phosphatase	70 units/L	40-130 units/L
Total Bilirubin	0.9 mg/dL	0.1-1.2 mg/dL
Albumin	4.5 g/dL	3.5-5.0 g/dL
Gamma-Glutamyl Transferase	35 units/L	9-48 units/L
Renal Function Tests		
Blood Urea Nitrogen	14 mg/dL	7-20 mg/dL
Creatinine	0.9 mg/dL	0.6-1.2 mg/dL
Glomerular Filtration Rate Estimate	>90 mL/minute/1.73 m²	>60 mL/minute/1.73 m²
Electrolytes		
Sodium	140 mEq/L	135-145 mEq/L
Potassium	4.2 mEq/L	3.5-5.1 mEq/L
Chloride	102 mEq/L	98-106 mEq/L
Bicarbonate	24 mEq/L	22-29 mEq/L
Inflammatory Markers		
C-Reactive Protein	45 mg/L	<5 mg/L
Erythrocyte Sedimentation Rate	55 mm/hour	0-20 mm/hour
Tumor Markers		
CA-125	21 U/mL	0-35 U/mL
Carcinoembryonic Antigen	2.5 ng/mL	0-5 ng/mL
Autoimmune Screening		
Antinuclear Antibodies	Negative	Negative
Rheumatoid Factor	Negative	Negative
Human Immunodeficiency Virus	Negative	Negative
Hepatitis B Surface Antigen	Negative	Negative
Hepatitis C Antibody	Negative	Negative

**Figure 1:**
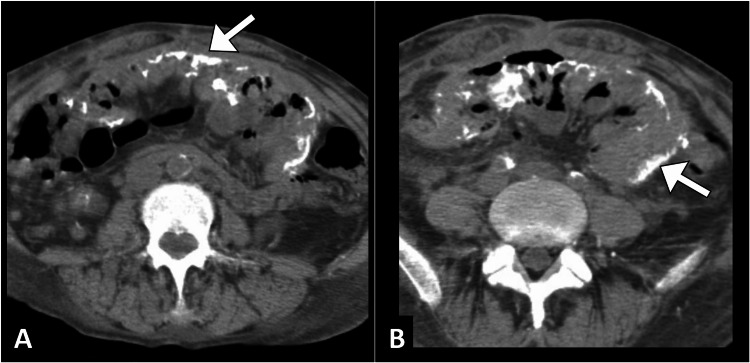
Axial CT images of the abdomen (A and B) demonstrating the encapsulation of small bowel loops by a calcified fibrous membrane (arrows), revealing the characteristic "cocoon-like" appearance indicative of sclerosing encapsulating peritonitis CT: computed tomography

Computed tomography of the abdomen and pelvis with a contrast agent demonstrated a "cocoon-like" encapsulation of the small bowel loops with associated ascites and peritoneal thickening, suggestive of sclerosing encapsulating peritonitis. There was no evidence of an intra-abdominal mass or lymphadenopathy (Figure 1).

The differential diagnosis for this patient included peritoneal carcinomatosis, tuberculosis peritonitis, chronic peritoneal dialysis-related encapsulating peritoneal sclerosis, and idiopathic sclerosing encapsulating peritonitis. Given the negative comprehensive workup for infectious, malignant, and autoimmune causes and the absence of a history of peritoneal dialysis, the diagnosis of idiopathic sclerosing encapsulating peritonitis was made.

Management involved a multidisciplinary approach involving gastroenterology, surgery, and nutrition. The patient was initially managed conservatively with total parenteral nutrition to improve her nutritional status and broad-spectrum antibiotics to cover any secondary infection. Due to persistent bowel obstruction symptoms and the failure of conservative management, surgical intervention was considered. The patient underwent an exploratory laparotomy, which revealed a thick fibrous capsule encapsulating the small bowel. Careful dissection and removal of the fibrotic capsule were performed, and the encapsulated bowel loops were released.

The postoperative course was uneventful, and the patient showed significant improvement in her symptoms. She was started on a gradual oral diet and was discharged on the 10th postoperative day. On follow-up visits over the next six months, the patient reported complete resolution of her abdominal pain and distension, and she had gained 5 kg in weight. The patient was closely monitored for any signs of recurrence, and regular follow-up appointments were made.

## Discussion

The case of idiopathic sclerosing encapsulating peritonitis presented herein adds to the scant but growing body of literature surrounding this rare and enigmatic condition. Sclerosing encapsulating peritonitis, or abdominal cocoon syndrome, is characterized by thick fibrous encapsulation of the small bowel, leading to symptoms of intestinal obstruction [1]. Although the pathogenesis of sclerosing encapsulating peritonitis is poorly understood, its association with peritoneal dialysis, abdominal surgery, tuberculosis, and certain autoimmune conditions suggests a multifactorial etiology involving both acquired and inherent predispositions [2-4]. The idiopathic form of sclerosing encapsulating peritonitis underscores the diagnostic and therapeutic challenges inherent to conditions with obscure etiologies.

The clinical presentation of sclerosing encapsulating peritonitis is notably nonspecific, often mirroring other more common causes of intestinal obstruction [2,3]. This nonspecificity, coupled with the rarity of the condition, frequently results in significant diagnostic delays. In the present case, extensive investigations were undertaken to exclude infectious, malignant, or autoimmune causes, highlighting the necessity of a thorough and systematic approach to differential diagnosis in patients with unexplained abdominal symptoms.

Imaging, particularly computed tomography, plays a pivotal role in the diagnosis of sclerosing encapsulating peritonitis, as evidenced in this case by the identification of the characteristic "cocoon-like" encapsulation of the small bowel [3-5]. These imaging modalities not only facilitate diagnosis but also aid in the planning of surgical intervention by delineating the extent of peritoneal involvement [4,5]. Despite the utility of imaging, the definitive diagnosis of sclerosing encapsulating peritonitis often requires surgical exploration, as was necessary in this case.

The management of sclerosing encapsulating peritonitis remains controversial, with options ranging from conservative approaches, including nutritional support and anti-inflammatory medications, to surgical intervention [2-4]. Surgical management, typically involving the removal of the fibrous capsule, is indicated in patients with intestinal obstruction or failure of conservative treatment [4,5]. The successful surgical management of the patient, followed by an uneventful recovery and significant symptomatic relief, emphasizes the potential benefits of surgical intervention in appropriately selected cases [5].

The long-term management of sclerosing encapsulating peritonitis involves monitoring for recurrence, which can occur and may necessitate further intervention [3,5]. The absence of recurrence in the present case over a six-month follow-up period is encouraging but underscores the need for ongoing vigilance.

## Conclusions

In conclusion, this case contributes to the understanding of idiopathic sclerosing encapsulating peritonitis and underscores several critical points. First, the findings highlight the importance of considering sclerosing encapsulating peritonitis in the differential diagnosis of unexplained abdominal symptoms, particularly in patients without a history of peritoneal dialysis or other known risk factors. Second, this study reinforces the role of comprehensive imaging in the diagnosis and preoperative planning of sclerosing encapsulating peritonitis. Finally, these findings support the utility of surgical intervention in patients refractory to conservative management while also emphasizing the necessity of long-term follow-up for the detection of recurrence.
